# Optimal Differentiation of In Vitro Keratinocytes Requires Multifactorial External Control

**DOI:** 10.1371/journal.pone.0077507

**Published:** 2013-10-07

**Authors:** Anne-Sophie Borowiec, Philippe Delcourt, Etienne Dewailly, Gabriel Bidaux

**Affiliations:** 1 Inserm U1003, Equipe Labellisee par la Ligue Nationale Contre le Cancer, Université Lille 1, Villeneuve d’Ascq, France; 2 Laboratory of Excellence, Ion Channels Science and Therapeutics, Université Lille 1, Villeneuve d’Ascq, France; Università degli Studi di Milano, Italy

## Abstract

For almost 30 years, keratinocyte differentiation has been studied in numerous cell models including keratinocyte primary culture with various supplemented culture media. In this respect, it has become quite difficult to draw comparisons between studies using such a variety of culture conditions. Serum-free condition with low calcium has been used to culture basal proliferating cells, though differentiation is induced by various procedures. These latter include the addition of calcium at mM concentration and a concomitant addition of serum and calcium. Lowering the incubation temperature of cells has also been reported to induce a premature differentiation of keratinocytes in organotypic skin culture. This effect of temperature on keratinocyte differentiation has been poorly depicted, although average human skin temperature has been shown to be about 32°C. However, studying differentiation and quantifying shifts in the differentiation rate of a cell population implies to precisely know i) the proportion of differentiated cells in the whole population, and ii) to which extent and to which level of expression, the induction of a gene or a protein might be considered as a marker of differentiation. This lack has rarely been taken into consideration and has surely led to over-interpretations of single protein induction and to consequent extrapolations to real differentiation processes. By means of paralleled analyses with immunocytofluorescence, flow cytometry, and with multiple differentiation markers quantify by qPCR and western-blot, we studied the paradoxical connection between calcium, serum, multilayer culture and incubation temperature on the differentiation of in vitro keratinocytes. Conversely to previous reports, we have shown that calcium switch is indeed a potent model for inducing calcium-dependent genes, but is not an efficient procedure when one wishes to assess the keratinocyte differentiation rate. Moreover, we have demonstrated that a synergic stimulation by calcium, serum, confluence and lower incubation temperature amplified the differentiation rate.

## Introduction

Epidermis is the outer compartment of skin and is formed by stratified layers of keratinocytes from the basal proliferating compartment up to the spinous, the granular and the cornified layers. For almost 30 years, keratinocyte differentiation has been studied with numerous cell models including orthotypic culture [1,2] and primary keratinocyte culture. In order to simplify culture procedures, the latter has been developed by removing the fibroblast-based feeder layer of a stratified culture. The mono-layered primary culture of keratinocytes has also let researchers to experiment on living cells. However, for many years now, we have been observing an increasing diversity in culture media (MCDB153, EpiLife, KSFM or KGM-2), as well as the development of serum-free culture supplements with various products, such as bovine pituitary extract (BPE) [3], calcium (Ca^2+^), epidermal growth factor, insulin, vitamin D [4] or vitamin C [5]. In this respect, it has become quite difficult to make comparisons between the studies using such a variety of culture conditions. Although low Ca^2+^, serum-free conditions have been widely used to culture basal proliferating cells, regarding – literature, differentiation may be induced with various other procedures. Indeed, one can use a Ca^2+^ [3] or serum/Ca^2+^ switch [6,7], or cell confluence [8]. It has thus become easy to get lost in this jungle of procedures and to formulate wrong interpretations.

Ca^2+^ has been shown to be a major factor in controlling keratinocyte differentiation [9]. It can sometimes be read here and there that the Ca^2+^ gradient increases linearly from the basal to the corneocyte layer, but over the last 30 years, several teams have demonstrated with different techniques that Ca^2+^ is distributed as a non-linear gradient in human skin with a low concentration in the basal layer, the lowest concentration being in the supra-basal / early spinosum layers and with a marked increase in the late spinosum, granular and corneosum compartments [10–12]. In recent years, a simplified consensus has appeared such as to the use of low Ca^2+^ (<0.1mM) supplements for proliferating and high Ca^2+^ (> 1mM) supplements for inducing differentiation. Strikingly, an original article demonstrated that optimal rate of keratinocyte proliferation was reached at about 0.3 mM Ca^2+,^ but dropped at lower Ca^2+^ concentrations (<0.1mM Ca^2+^) in serum free conditions [3], thereby coinciding with the endogenous gradient of Ca^2+^ in human epidermis.

The induction of keratinocyte differentiation is even more confusing. Indeed, authors have described a permissive effect of high Ca^2+^ on differentiation marker expression [7,13], whilst others have shown a high-Ca^2+^ mediated induction of differentiation markers [14,15]. Serum has been reported to trigger proliferation arrest and to induce the differentiation of mouse primary keratinocytes [2,6,16]. Besides, a recent study showed that culturing human keratinocytes in serum-free condition for several passages made them unable to form stratified layers in reconstituted skin model [17]. Paradoxically, it has been suggested the vitamin A contained in serum did counteract differentiation of keratinocytes [18] and to repress keratin 10 expression. Consequently, serum-free media were developed and the BPE supplementation has become a standard in serum-free media, since it increases basal keratinocyte proliferation and increases their survival. It may also be noted that BPE inhibits terminal differentiation partially [3]. Furthermore, it is accepted that the differentiation of granular-like keratinocytes is achieved in confluent, multilayer keratinocyte cultures [8,14,15], whiles others have demonstrated that terminal differentiation could be achieved in detached isolated keratinocytes [7]. Finally, it has been shown that culture at the air-liquid interface is required for cornified barrier formation in orthotypic cultures [19].

A further interesting feature of human skin is its exposure to large variations in temperature. At a comfortable external temperature (about 30°C), average physiological human skin reaches about 32°C [20]. Surprisingly, almost all the studies conducted on cultured keratinocytes have been performed at 37°C. To our knowledge, only one group studied the effect of incubation at 32°C on the proliferation/differentiation balance of keratinocytes. Indeed, Ponec et al demonstrated that the de-epidermized dermis regenerated a well-differentiated epidermis with a morphology showing a higher resemblance to the native epidermis than the cultures grown at 37°C [21,22]. In addition, it has recently been reported that 31°C was optimal for ATP release by keratinocytes [23]. Because, ATP has been thought to be a potent autocrine factor in the regulation of the proliferation/differentiation balance [24], lower temperature may optimize differentiation of keratinocytes.

Quantifying shift in the rate of differentiation of a cell population implies a prior characterization and quantification of keratinocyte phenotypes (basal, spinal-like, granular-like, corneocytes), defined with the use of molecular markers. Consequently the qualitative and quantitative expression of these molecular markers has to be determined in the cell models and compared to the ones of in vivo skin. However, this very equivalence has rarely been taken into consideration and has probably led to over-interpretations considering that single protein induction may be extrapolated as an increase of the differentiation rate.

Therefore, in this study, we developed a set of differentiation markers beyond those classic secondary filament markers, in order to discriminate between single gene induction and the wide rearrangement of gene/protein expression expected to occur during differentiation. We indeed highlighted the limits of qPCR and immunoblotting (IB), but also showed the advantages of making parallel analyses with immunocytofluorescence (ICF) and flow cytometry (FC), when studying variations in the differentiation rate. Regarding these technical issues, we studied the paradoxical connection between calcium, serum, multilayer culture, and incubation temperature on the differentiation of in vitro keratinocytes. Unlike some former reports, we demonstrated that calcium switch is indeed a potent model for inducing calcium-dependent genes, but is not an appropriate procedure for assessing keratinocyte differentiation. In addition, we demonstrated that a synergic stimulation by calcium, serum, confluence and a lower incubation temperature accelerated the differentiation rate.

## Materials and Methods

### Cell culture

HaCaT cell line was grown in Dulbecco’s minimal essential medium, DMEM, (Gibco) supplemented with 2% fetal calf serum (FCS), Kanamycin (100 µg/ml), Sodium Pyruvate (1mM) and Ca^2+^ was adjusted to 0.2 mM for basal cell culture. Ca^2+^ was adjusted to a final 1.8 mM to induce differentiation. hNEK cells were obtained from Lifescience Inc. and plated in 10 cm round dishes in basal KSF-SFM medium at a density of 250 k cells/dish. Basal KSF-SFM medium was supplemented with bovine pituitary extract, EGF, glutamine and kanamycin as advised by the manufacturer (Invitrogen). 2% FCS or Ca^2+^ adjusted to 1.8 mM were added to basal KSF-SFM media to induce keratinocyte differentiation.

### Primary culture of mouse keratinocytes (mPK)

This study was carried out in strict accordance with the recommendations in the Guide for the Care and Use of Laboratory Animals of the French National Institute for Medical Research (INSERM). The Committee on the Ethics approved the protocol for Animal Experiments of University of Lille 2 (Permit number: CEEA 08/2009). All efforts were made to minimize suffering.

After sacrifice, the backskins of C57BL/6J mice were shaved with an electric razor prior to skin removal. The isolation and culture protocol for epithelial cells was derived from Nowak and Fuchs [25]. The skin was set hair side up on the dissecting pad. Using the scalpel, the fat and blood vessels covering the dermis were scraped until the dermis was clearly and uniformly exposed. Then the skin, dermis side down, was put in solution with 0.25% trypsin, making sure that the skin was freely floating with an unsubmerged epidermis. The skin was incubated overnight at 4°C. The following day, using a scalpel and forceps we separated the epidermis from the dermis by scraping along the top to remove the epidermis.

The dermis was removed from the dish and the largest pieces of epidermal tissue were briefly minced. After pipetting up and down to triturate the tissue, Defined-keratinocyte medium (Gibco) containing 10% FCS was added to inactivate the trypsin. The epidermis mixture was then passed through a 70 µm cell strainer. After washing the strainer with 5 ml of medium, the cell suspension was centrifuged for 10 min at 250 g. Epidermal cells were then re-suspended in fresh Defined-keratinocyte medium without serum and grown in adapted petri dishes. To avoid fibroblast development, cells were cultured in this medium for 5 days. They then were grown in basal KSFM medium to increase cell growth.

### Quantitative real-time PCR analysis (qPCR)

After total mRNA extraction and purification with TRI REAGENT^®^ (Sigma-Aldrich), mRNA were subjected to DNAse treatment (Ambion) at 0.25µl DNAse per µg of RNA for 25 min at 25°C. Afterwards, 10 µg mRNA were purified in a V/V phenol/chloroform/AIA solution (Fluka) with 5% Sodium Acetate 3M. The upper phase was supplemented with 10% Sodium Acetate 3M and 2.5 V 100% Ethanol and kept at -20°C overnight in order to precipitate. After a brief wash in 70% Ethanol, pellets were left to dry and then re-suspended in 30 µl water. After an agarose gel check of mRNA quality, 2µg of mRNA were subjected to reverse transcription as reported elsewhere [26]. Real-time quantitative PCR was performed on a Cfx C1000 system (Biorad). For each reaction, 12.5 ng of cDNA was placed in a final reaction mixture of 15µl containing 7.5µl of 2x SsoFast™ EvaGreen^®^ Supermix (Biorad) and 200nM primer pairs (see Table 1). The PCR protocol was: an initial 30 sec denaturation step at 95°C, and 40 cycles of [4 sec at 95°C, 30 sec at 60°C] and a final dissociation curve to control the specificity of the amplification.

**Table 1:** Oligonucleotides used to perform qPCR.

	Forward	Reverse
Keratin 1	ATTTCTGAGCTGAATCGTGTGATC	CTTGGCATCCTTGAGGGCATT
Keratin 10	TGATGTGAATGTGGAAATGAATGC	GTAGTCAGTTCCTTGCTCTTTTCA
Keratin 16	TGCCCACCTTTCCTCCCAGCAA	CCGGGTCTGACGGCTCGAAG
Keratin 5	CTGCTGGAGGGCGAGGAATGC	CCACCGAGGCCACCGCCATA
Keratin 14	TGGACGTGAAGACGCGGCTGG	GATTTGGCGGCTGGAGGAGGTC
Filaggrin	CTGGACACTCAGGTTCCCAT	TTTCGTGTTTGTCTGCTTGC
Involucrin	CTGCCTCAGCCTTACTGTGA	GGAGGAGGAACAGTCTTGAGG
GAPDH	ACCCACTCCTCCACCTTTG	CTCTTGTGCTCTTGCTGGG
Transglutaminase 1	TCACTGTTTCATTGTCTCCA	CCCTCACCAATGTCGTCTTC
PCNA	CGACACCTACCGCTGCGACC	TAGCGCCAAGGTATCCGCGT
CDKN1B	AGCGGAGCAATGCGCAGGAA	GGCGTCTGCTCCACAGAACCG
CDKN1A	TCAGGGTCGAAAACGGCGGC	TTTGAGGCCCTCGCGCTTCC
EIF2AK3	GCTGTCGGACCTCGCAGTGG	TCCGGCTCTCGTTTCCATGTCTG
ATF4	TCAGGGTCCACGGCCACCAT	ACGCTGCTGCTGAATGCCGT
DDIT3	AAAGATGAGCGGGTGGCAGCG	AGCTGCCATCTCTGCAGTTGGAT
P2RX7	GACCGAGGTTGTAAAAAGGG	ACCAGGCAGAGACTTCACAG
P2RY2	AGCTCTTCAGCCGCTTCG	GCTTTAGCAGTCGCCGA
18S	CAGCTTCCGGGAAACCAAAGTC	AATTAAGCCGCAGGCTCCACTC
HPRT	GGCGTCGTGATTAGTGATGAT	CGAGCAAGACGTTCAGTCCT
ATF6	TGAAGCCATCCGCAGAAGGGGA	GGGTGGTAGCTGGTAACAGCAGG
DNAJ	AGAACGCTCGGTGAGAGGCGG	CGGTGTGTGAGGGAGCGGGAA

The housekeeping gene Glyceraldehyde-3-phosphate dehydrogenase (GAPDH) was used as an endogenous control to normalize variations in RNA extractions, the degree of RNA degradation and variability in RT efficiency. GAPDH mRNA has been selected as the most invariant gene in a set of 6 reporters: HPRT mRNA, GAPDH mRNA, 18S rRNA, ATF6 mRNA, DNAJ mRNA and KRT17 mRNA. Mean Ct were calculated for the four experimental procedures of cell culturing: (-Ca^2+^, -FCS), (+Ca^2+^, -FCS), (-Ca^2+^, +FCS) and (+Ca^2+^, +FCS) and Variances of mean Ct were figured out for each mRNA in order to determine the invariant. Variances of 18S rRNA, HPRT mRNA, GAPDH mRNA, ATF6 mRNA, DNAJ mRNA and KRT17 mRNA were respectively: 2.57, 0.1, 0.25, 0.22, 0.46 and 0.68. We used the comparative Ct method to quantify mRNA levels.

### Immunoblotting

An ice-cold buffer (pH 7.2) containing 10 mM PO4Na2/K buffer, 150 mM NaCl, 1 g/100 ml sodium deoxycholate, 1% Triton X-100, 1% NP40, a mixture of protease inhibitors (Sigma-Aldrich), and a phosphatase inhibitor (sodium orthovanadate; Sigma-Aldrich) was applied to previously PBS-washed cells in dishes. After 30min incubation on ice, the protein extract was transferred to 1.5 ml tubes and subjected to sonication. After 10 minutes of centrifugation at 15,000 g, the pellet was transferred into a clean tube prior to a determination of the protein concentration using a BCA Protein Assay (Pierce). 25 µg of total protein were loaded onto a 10% polyacrylamide gel before an SDS-page was performed. After electrophoresis, proteins were transferred to a nitrocellulose membrane using a semi-dry electroblotter (Bio-Rad). The membrane was blocked in a TNT +5% (W/V) milk (15 mM Tris buffer, pH 8, 140 mM NaCl, 0.05% Tween 20, and 5% non-fat dried milk) for 30 min at room temperature, then soaked in primary antibody diluted in TNT +1% milk for either 2 h at room temperature or overnight at +4°C. After three washes in TNT, the membrane was soaked in secondary antibody diluted in TNT+1% milk for 1h at room temperature. The membrane was processed for chemiluminescence detection using Luminata Forte Western HRP Substrate (Millipore) according to the manufacturer’s instructions. After a 10 min bath in Re-blot PLus Mild SOlution (Millipore), membrane was blotted again. The primary antibodies used were: anti-Keratin 5 (Covance) at 1/2000, anti-Keratin 10 (Covance) at 1/1000, anti-Involucrin (Sigma) at 1/500, anti-Filaggrin (Abcam) at 1/200, anti-Vimentin (Santa Cruz) at 1/400, anti-GAPDH (Santa Cruz) at 1/250. Secondary antibodies were coupled to either green fluorochrome DyeLight-488 (Molecular probes) or red fluorochrome AlexaFluor-546 (Molecular probes). Nuclei were counterstained with Dapi (1/400, Dako).

### Immunofluorescence

Keratinocytes were plated on 35 mm glass bottom dishes (MatTek Inc) and grown in the desired medium. Anonymous human skin resections specimens were obtained after breast reduction surgery according to the Declaration of Helsinki Principles and the guidelines of ethical committee of the Regional Hospital (CHRU Lille). The ethical committee of the General Hospital (CHRU Lille) approved the collection and storage of this tissue for future research. Tissue donors have been informed and write consent to have their tissue stored and used for future research. Frozen sections and cells were fixed with 4% formalin in PBS for 10 min on ice prior to 3 PBS washes. Frozen sections of human breast Skin (10 µm thick) or cells were subjected to blocking and permeabilization with PBS + 1.2% gelatine + 0.2% Tween + 0.2M glycine for 30 min at 37°C. Frozen sections of human breast skin were directly subjected to this blocking step. The slides/dishes were then incubated with primary antibodies 2 h at 37°C. After thorough rinsing in PBS/gelatine, the slides/dishes were treated with the corresponding secondary antibody: either Dye light 488-labeled anti-rabbit IgG (Jackson ImmunoResearch; dilution, 1/2000) or Alexa fluor 546-labeled anti-mouse IgG (Molecular Probes; dilution, 1/4000) diluted in PBS/gelatine for 1 h at room temperature. After rinsing twice in PBS/gelatine and once in PBS with 1/200 Dapi for 10 min at RT, the slides were mounted with Mowiol® and examined under a confocal microscope. The primary antibodies used were: anti-Keratin 5 (Covance) at 1/2000, anti-Keratin 10 (Covance) at 1/100 and anti-Keratin 10 (Abcam) at 1/200, anti-Involucrin (Sigma) at 1/1000 and anti-Involucrin (Abcam) at 1/400, anti-Filaggrin (Abcam) at 1/800, anti-Loricrin (Abcam) at 1/1000.

### Flow cytometry

Flow cytometry was performed with a CyAn™ ADP Analyser. Cells were harvested, split into 1 million cell samples in 15 ml tubes before fixation with 1ml of 70% Ethanol at -20°C overnight. Cells were washed twice with PBS / 4% BSA / 0,1% TritonX100 and finally incubated at RT for 30 min. Primary antibodies were diluted in 100 µl PBS-BT at the same dilution as that for immunofluorescence and incubated with cells at RT for 1h. After a first quick wash in PBS, a second wash was done at RT for 30 min. Secondary antibodies: anti-rabbit IgG coupled to DL-488 and anti-mouse IgG coupled to AF-647 were diluted respectively at 1/2000 and 1/4000 and incubated with cells at RT for 30 min. Afterwards, cells were washed out 3 times at RT for a total of 30 minutes. The flow cytometer was calibrated with rainbow beads before each experiment. 488 and 642 lasers were used. Data were analyzed with FlowJo software (v 8.7).

### Confocal microscopy

Confocal imaging experiments were performed using a confocal laser scanning microscope (LSM 780; Carl Zeiss MicroImaging, Inc) and a plan-Apochromat 40×/1.3 NA oil immersion objective. Parameters were fixed once and used for acquiring all the conditions in order to carry out quantitative analyses (Parameters 1): pinhole: 1.7 µM, laser current _(488nM)_: 1.1 A, gain_(488nM)_: 740, offset_(488nM)_: 0, laser current _(546nM)_: 3 A, gain_(546nM)_: 700, offset_(546nM)_: -5. A second set of parameters was used to compare level of expression of keratin 10 in human skin sections and in human cultured keratinocytes, and to study the polymerization of the K10 network in cultured keratinocytes. Parameters 2 were: pinhole: 0,9 µM, laser current _(488nM)_: 0.5 A, gain_(488nM)_: 720, offset_(488nM)_: 0, laser current _(546nM)_: 1 A, gain_(546nM)_: 700, offset_(546nM)_: -5. For z-scanning and 3D deconvolution images, parameters were adjusted to parameters 2. Images were acquired with ZEN software (Carl Zeiss MicroImaging, Inc) and data analyses were achieved with ImageJ64 freeware.

### Data analysis

Each experiment was repeated at least three times and the results were expressed as Mean ± S.D. Data were analysed and graphs plotted using either Origin 5.0 software (Microcal, Northampton, MA) or Excel. InStat3 (GraphPad Software Inc, SanDiego, USA) was used for statistical analysis and the mean values were compared using either an unpaired t test with Welch’s corrected test (2 groups) or One-way ANOVA with Dunnett’s multiple comparison post-test (≥ 3 groups). Statistical significances were: * = p<0.05; ** = p<0.01; *** = p<0.001.

## Results

### Serum (FCS) and calcium (Ca^2+^) switch increase expression of a large scale of differentiation markers

**Figure 1:**
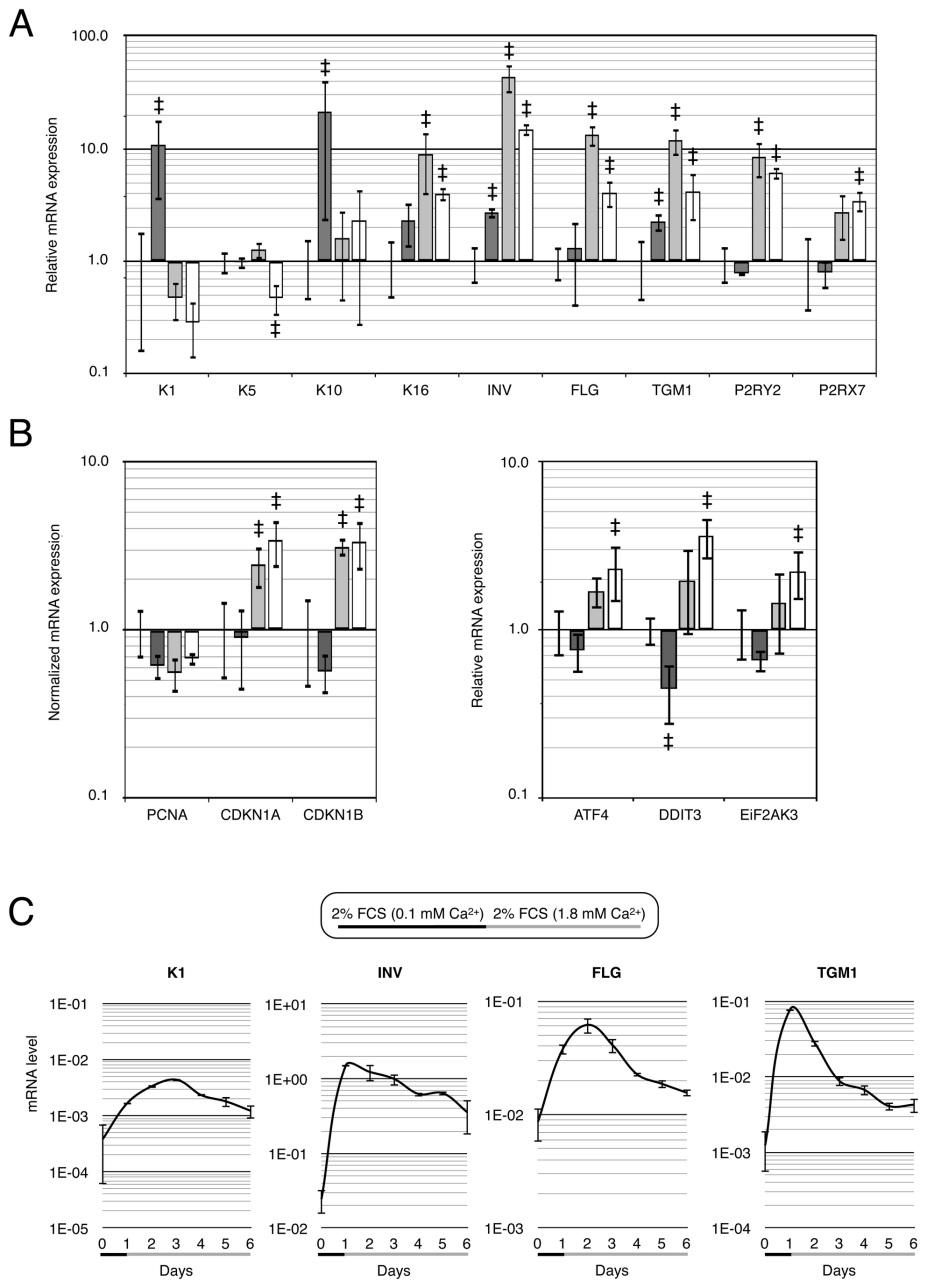
Analysis of gene expression reveals major rearrangement after Ca^2+^/FCS induction. Serum and calcium-dependent genes in 60%-confluent hNEK cultures. A. Histogram represents normalized mRNA levels determined by qPCR in cells cultured in basal medium (no FCS, 0.1 mM Ca^2+^) reports with black columns, or after a 3-day calcium switch (no FCS, 1.8 mM Ca^2+^) reports with dark grey column, or after a 3-day serum switch (2% FCS, 0.1 mM Ca^2+^) reports with light grey columns or after a 3-day double serum/calcium switch (2% FCS, 1.8 mM Ca^2+^) reports with white columns. Genes studied were: 1) markers of basal epidermal compartment: Keratin 5 (K5) and P2Y purinoceptor 2 (P2Y2), 2) marker of proliferative epidermal compartment: Keratin 16 (K16), 3) markers of spinal compartment: Keratin 1 (K1) and 10 (K10), 4) markers of granular compartment: Involucrin (INV), Filaggrin (FLG), Transglutaminase (TGM1), P2X purinoceptor 7 (P2X7). mRNA level has been calculated as described in *Materiel* and *methods* for the four experimental conditions of cell culturing before its normalization on the value for the -Ca^2+^ /-FCS condition. Data are presented as Mean ± SD (N=6). Significance was reached when p< 0.05 (‡). B. Same as (A) for mRNA levels of proliferating cell nuclear antigen (PCNA) and the inhibitors of cell cycle p21 (CDKN1A) and p27 (CDKN1B), left panel, and ER-stress related genes: PERK (EiF2AK3), ATF4 and ER-stress effector CHOP (DDIT3), right panel (N=6). C. Kinetics of keratinocyte differentiation reveals different serum and calcium sensitivities of gene expression. Values represent mRNA levels of the genes of interest normalized with GAPDH mRNA levels. Confluent hNEK were grown in 2% FCS + 0.1 mM Ca^2+^ for 1 day prior to an addition of 1.8 mM Ca^2+^ for 5 days (N=4). All points were significantly different from Day 0 with p<0.001.

Differentiation of keratinocytes engages profound modifications of transcriptome and proteome that leads to changes of the cell phenotype. It is therefore a great challenge to discriminate between what a single gene induction is and what constitutes a global rearrangement of gene expression associated with the differentiation process. We hypothesized that the detection of multiple markers from different gene families would be a most efficient way to report differentiation instead of a limited number of cytoskeleton-related genes, as usually performed. Because there is still a strong controversy as to the fundamental role of both Ca^2+^ and serum in inducing keratinocyte differentiation, we firstly aimed to bring elements to the debate. Non-confluent human normal epidermal keratinocytes (hNEK) cultured in a basal medium (no Fetal Calf Serum, FCS, and 0.07 mM Ca^2+^) were induced to differentiate with a 3-day Ca^2+^ switch (1.8 mM Ca^2+^), or a 3-day serum switch (2% FCS) or a 3-day Ca^2+^/FCS switch (1.8 mM Ca^2+^ + 2% FCS) and proceeded to gene expression analysis with qPCR. Note that the *Glyceraldehyde 3-phosphate dehydrogenase* (GAPDH) gene was sorting out as one of the most invariable genes in this experiment (see Materiel and Methods for further explanations), and was then used to normalize the expression of genes of interest. As reported in Figure 1A, the Ca^2+^ switch triggered an induction of pro-proliferative Keratin 16 (K6), of supra-basal Keratin 1 (K1) and Keratin 10 (K10), and P2Y purinoceptor 2 (P2YR2) [27,28]. It also induced slightly (a 2 to 4 fold increase) the late differentiation markers, Involucrin (INV) and transglutaminase (TGM1), but modified neither Filaggrin (FLG) nor the P2X purinoceptor 7 (P2RX7). The FCS switch produced a decrease in K1, induced high levels of K16 (9 fold), INV (40 fold), TGM1 (15 fold), FLG (15 fold), P2RY2 (8 fold) and P2XY7, but had no effect on K10. The Ca^2+^/FCS switch presented an effect similar to that of the FCS switch alone, but with a decrease in basal marker Keratin 5 (K5) expression. We also noted that, in accordance with a previous study [13], Ca^2+^ exerted a moderate permissive effect on INV, FLG and TGM1 in the presence of 2% FCS.

The differentiation of keratinocytes has been associated with proliferation arrest [3,14] characterized by an induction of *CDKN1A* and *CDKN1B* genes encoding respectively the cell cycle inhibitors p21^waf1^ and p27^cip^ [29,30]. We measured the expression of cell cycle markers PCNA, CDKN1A, CDKN1B, yet we failed to detect any significant variation in the PCNA level which was expected to decrease whether cells entered G0 phase [31]. However, we did detect increases in CDKN1A, CDKN1B mRNA levels after the FCS switch and the Ca^2+^/FCS switch (Figure 1B, left panel), thereby suggesting that FCS exerted an anti-proliferative effect by controlling the key inhibitors of G1/S and G2/M checkpoints. In addition, Ca^2+^ switch failed to modify either PCNA, p21 or p27 expression, which would infer that the reported anti-proliferating effect of high Ca^2+^ levels is unlikely related to cell cycle inhibitors.

Although keratins, transglutaminases and intermediate filament proteins such as LR, FLG are the most commonly used differentiation markers, it has also been reported that keratinocyte differentiation involves ER stress through an “Unfolding Protein Response” [32]. We therefore assessed the expression of genes involved in induction of the ER stress response (PERK, ATF4) and of the ER stress integrator, Chop [33]. A Ca^2+^ switch was found to reduce DDIT3 expression (Chop), suggesting that no further ER stress occurred (Figure 1B, right panel). An FCS switch did not significantly modify the expression of these genes either. Contrastingly, the combined Ca^2+^/FCS switch significantly increased the expression of ATF4, DDIT3 and EiF2AK3. Next, we wondered whether the permissive effect of Ca^2+^ on the FCS switch could be time-dependent. hNEK were plated at 90% confluence in basal KSFM supplemented with 2% FCS for 24h. In order to limit confluence-mediated differentiation, the Ca^2+^ switch was achieved on confluent cell cultures at D1 (Figure 1C). As shown in Figure 1C, FCS strongly induced INV, TGM1 and FLG gene expression, before Ca^2+^ partially repressed *INV* and *TGM1* genes but transiently induces *FLG*. In contrast, K1 was induced transiently with a peak expression 2 days after the Ca^2+^ switch, suggesting a positive but transient effect of a Ca^2+^ on FCS switch. CDKN1A, CDKN1B and P2RX7 genes were up regulated by a FCS switch but unaffected by a Ca^2+^ switch, although pro-proliferative P2RY2 expression decreased slowly 2 days after the Ca^2+^ switch (Figure S1). Note that the mRNA levels decreased after a 3-to-4 day-induction, though they reached a significantly higher level than the one before induction. This demonstrates that switching the medium composition induces a transient gene expression and reaches a new equilibrium after 3-4 days.

Altogether, these data demonstrate that a Ca^2+^ switch induces a transient induction of K1 and K10 gene expression, but modifies neither the cell cycle key regulator expression nor the ER stress response induction. However, ER stress, an induction of cell cycle inhibitors and an induction of late differentiation markers occur after an FCS switch and are further increased by a sizeable addition of Ca^2+^.

**Figure 2:**
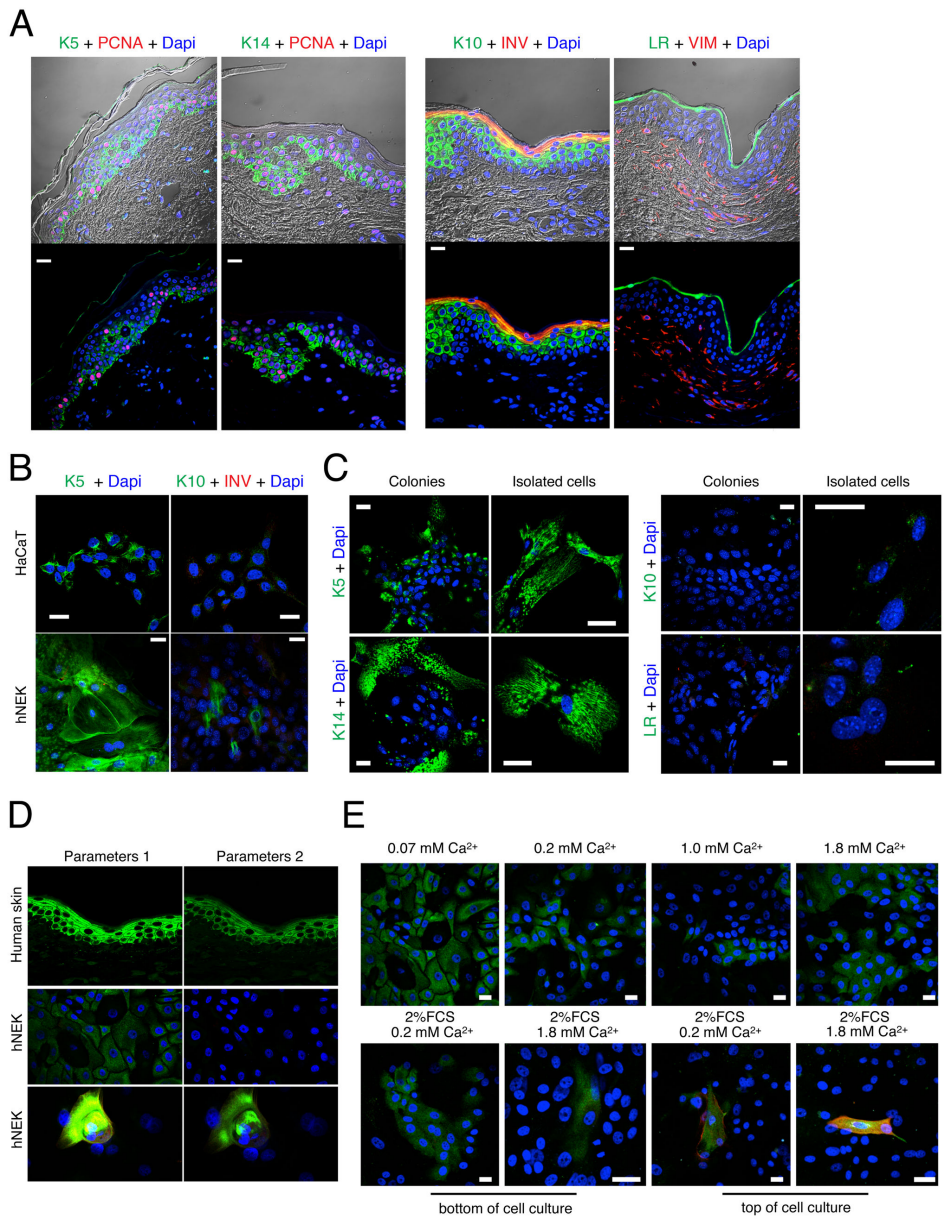
Molecular phenotypes of *in vitro* cultured keratinocytes diverge from *in situ* keratinocytes. Expression of markers of keratinocyte differentiation in human skin, HaCaT cell line, human Normal Epidermal Keratinocytes (hNEK) and Primary culture of mouse Keratinocytes (mPK). A. Images acquired with a LSM780 confocal microscope (Parameters 2, see *Material* and *Methods*) represent 0.9 µm thick slides of immunolabbeled human frozen skin sections. Panels show detection of 1) keratin 5 (green), K5, and PCNA (red), top left panel, 2) keratin 14 (green), K14, and PCNA (red), bottom left panel, 3) keratin 10 (green), K10, and Involucrin (red), INV, top right panel, 4) loricrin (green), LR, and vimentin (red), VIM, bottom right panel. B. Represents the immunodetection of K5 (green), K10 (green) or INV (red) in non-confluent HaCaT cells and hNEK cultured in media completed to 1.8 mM Ca^2+^. Images were acquired with Parameters 2. hNEK populations were not confluent though colonies were formed locally. C. The same as B for isolated and confluent mPK. K5, K10, K14 and LR are labeled in green. D. Panel shows the sensitivity of detection of K10 (green) with 2 different sets of parameters (parameters 1 and 2) of the confocal microscope. With parameters 1, the most sensitive, K10 shows a strong expression in human skin sections (top left panel), a low expression in the most part of hNEK (middle left panel) and a strong expression in a low population of hNEK (bottom left panel). With parameters 2, the less sensitive, K10 is only seen in human skin sections and in a low population of hNEK. E. Images of immunocytofluorescence show the detection of K10 and INV in hNEK cultured with increasing Ca^2+^ concentrations (top panels) or with 2% FCS at different Ca^2+^ concentrations (bottom panels). The confocal microscope was set with Parameters 1. All experiments were reproduced three times independently. Scale bars = 10 µm.

While differentiation markers (K10 and INV) are induced at mRNA level, concurrent protein induction is not mandatory [14]. We speculated that a population analysis (i.e. qPCR, immunoblotting) may hide a limited but differentiated keratinocyte population, but that single cell analysis (i.e. immunocytofluorescence, flow cytometry) should reveal it. Since culturing cell at confluence induces keratinocyte differentiation even at low extracellular Ca^2+^ levels, we studied both confluent and non-confluent keratinocytes with immunocytofluorescence. Firstly, we checked the specificity of antibodies in human skin (Figure 2A). As expected, both K5 and K14 were detected in the basal layer, PCNA positive proliferating cells, and in the suprabasal non-proliferating layer. K10 was detected at low level in the suprabasal, and at high levels in both spinosum and granulosum layers. As reported, INV was expressed in the late spinosum and granulosum compartments, while the granular marker LR, and the stromal marker Vimentin (VIM) were detected inside the granular layer of epidermis and inside the dermis respectively. Using identical microscope settings (Parameters 2, see *Materiel and Methods*) as in Figure 2A, we detected intense K5 and K14 signals in the HaCaT keratinocyte cell line grown in a DMEM medium completed with 2% FCS and subjected to 1.8 mM Ca^2+^ switch (Figure 2B). Surprisingly a very faint signal for K10 was detected, although no INV (Figure 2B), and no LR signals appeared (data not shown). hNEK (Figure 2B) and primary culture from mouse keratinocytes, mPK (Figure 2C), grown in basal KSFM prior to a 1.8 mM Ca^2+^ switch, show intense basal marker expressions with polymerized structures, some faint K10 expression in a small number of cells, and a very weak number, if significant, of cells were positive for INV or LR signals. These data demonstrate that about 99% of isolated or colonies-forming keratinocytes present either basal or suprabasal phenotypes, independently of their confluence status. Controversially to what has been reported previously, we did not found that a 3-day Ca^2+^ switch induced a massive keratinocyte differentiation, even though a moderate number of cells indeed showed expression of differentiation markers.

In order to detect the lowest levels of differentiation markers, we increased the sensitivity of the confocal microscope and compared K10 and INV signals acquired with two different settings (parameters 1 and 2, for details see *Materiel and Methods*). Increased sensitivity of detection revealed a discrete K10 expression in the main part of hNEK (Figure 2D, left panel) and a strong K10 and INV expression in a very low number of cells, which correspond to the K10 ^+^ /INV^+^ cells detected in the Figure 2B and 2C. As far as we know, this quantitative comparison of K10 and INV levels between immunolabeled keratinocytes and human skin sections has never been performed and demonstrate that the sensitivity of fluorescence microscope has to be carefully calibrated before claiming that i) a protein is indeed expressed, ii) phenotype of cultured cell represent of good model for interpreting tissue physiology. We next assumed that in previous studies, authors may have detected keratinocytes expressing low levels of K10 to study the Ca^2+^ switch effect and we performed a series of experiments using “Parameters 1”. Almost 80% of hNEK grown in basal KSFM medium presented a faint K10 expression, which decreased in correlation with the Ca^2+^ increase at 0.2 mM, and at 1 mM (Figure 2E, top panels). However, a 1.8 mM Ca^2+^ switch seemed to restore the percentage of K10^+^ cells. FCS addition in low Ca^2+^ also decreased the percentage of low-level-K10 positive cells, although a concomitant faint detection of K10 and INV is noted in a moderate number of cells. With the addition of 1.8 mM Ca^2+^ to a 2% FCS medium, we estimated that about 1% of cells, specifically located on the upper layer of the culture, expressed strong levels of both K10 and INV (Figure 2E, bottom panels).

The proportion of differentiated keratinocytes is related to the proportion of keratinocytes with the potency to differentiate. This latter excludes, *de facto*, others cell types, like fibroblasts, but also Epithelial-mesenchymal transition (EMT) keratinocytes, which are characterized by their potency to migrate. Fibroblast and, EMT-like keratinocytes grown in low-calcium conditions [34], express vimentin (VIM), Conversely to *in situ* keratinocytes (Figure 2A), K5 has been detected in about 95% of VIM^+^ cells (Figure S2A), while similar proportion of VIM^+^ keratinocytes has been observed in low and high calcium media (Figure S2B). However, we found the proportion of VIM^+^ cells (about 10%) increased in cells grown in either FCS-supplemented media. This concomitant expression of K5 and VIM in keratinocytes suggests that this cell population is not willing to differentiate. Consequently, in FCS-supplemented cultures, a decrease in the number of differentiating and differentiated cells could have been expected - conversely to what has been observed.

When considered together, our data suggests that a Ca^2+^ switch procedure is a good way of looking for Ca^2+^-dependent genes, but is not a potent tool for assessing differentiation. Conversely, a Ca^2+^/FCS switch induces about 1% of hNEK to differentiate in late spinal-like or granular-like keratinocyte phenotype.

### Slight decrease of temperature inhibits proliferation but stimulates differentiation

**Figure 3:**
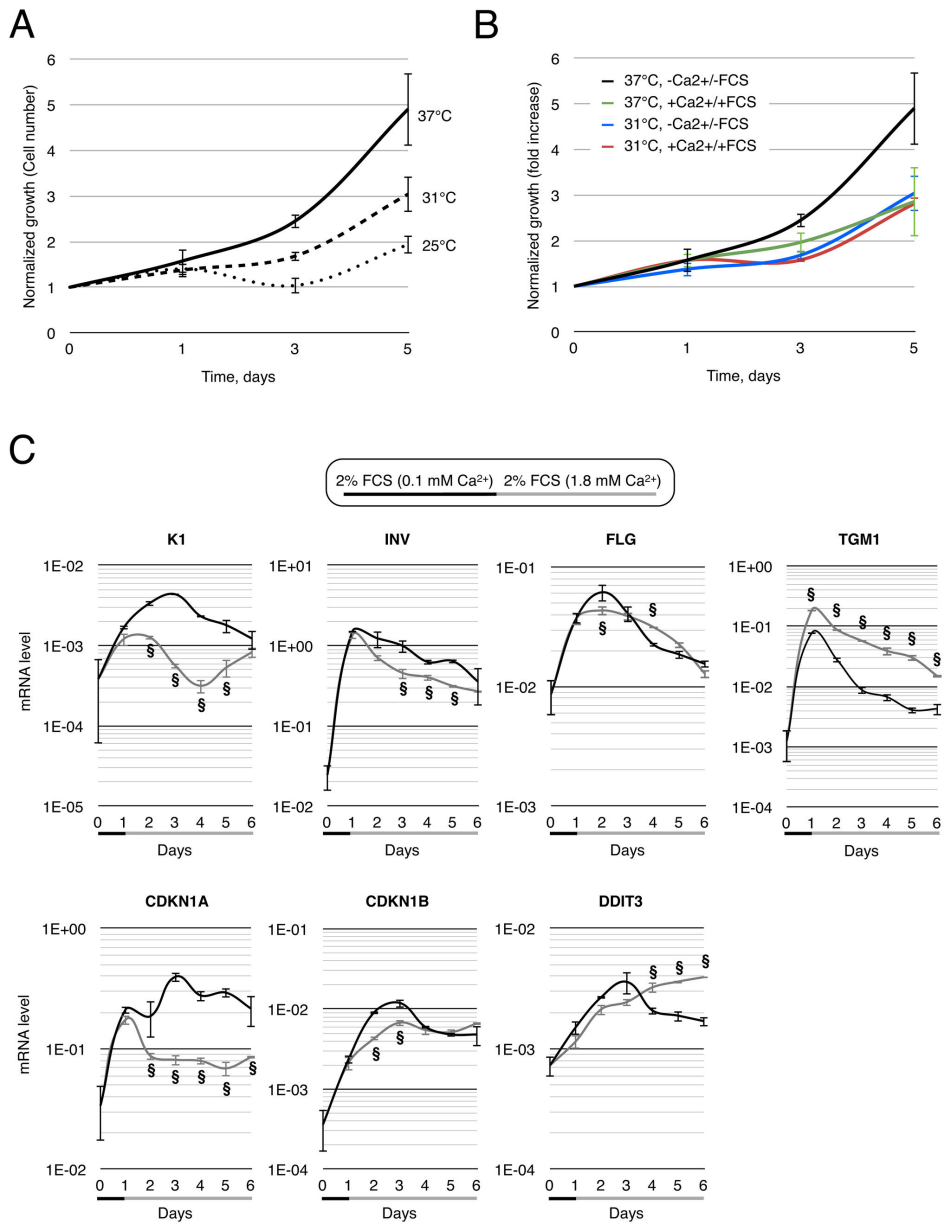
Lower incubation temperature modulates growth and gene expression in keratinocytes. A. Growth rate of hNEK cultured in basal medium at different incubation temperatures 37, 31 and 25°C was estimated by cell counting. Data are presented as Mean ± SD of three independent experiments. B. Growth rate of hNEK cultured either in basal (-Ca^2+^ /-FCS) or in 1.8 mM Ca^2+^ + 2% FCS complete media (+Ca^2+^ /+FCS) at 37°C or 31°C was estimated as in (A). C. mRNA quantification of keratin 1 (K1), involucrin (INV), filaggrin (FLG), transglutaminase (TGM1), p21^waf1^ cell cycle inhibitor (CDKN1A), p27^kip^ cell cycle inhibitor (CDKN1B) and, ER-stress reporter, Chop (DDIT3). hNEK were grown in 2% FCS + 0.1 mM Ca^2+^ for 1 day prior to the addition of 1.8 mM Ca^2+^ for 5 days (N=4). Kinetics of gene expression was assessed by real-time PCR for cells incubate either at 37°C (black line) or 31°C (grey line). Results are presented as Mean ± SD and are normalized for GAPDH expression. Significance was reached when p< 0.05 (§).

Infra-red thermography of the human body has demonstrated that the average epidermis temperature of a naked person is about 32°C at an ambient temperature of 30°C [20]. We wondered whether a mild cold incubation might alter the keratinocyte proliferation/differentiation balance. The decreasing of the incubation temperature at 31°C and 25°C gradually reduced the proliferation rate of both hNEK cultured in a basal medium (Figure 3A) and of a HaCaT cell line cultured in low Ca^2+^ medium (Figure S3A). This would likely by caused by the gradual decrease of enzyme activity at a temperature below the temperature of their optimal rate of activity. At 25°C, cell mortality had increased in the first 3 days of hNEK culture and had continuously increased in HaCaT cell culture, as shown by decreasing slopes. We also noted that FCS and Ca^2+^ reduced the proliferation rate of keratinocytes grown at 37°C, but did not further affect the growth rate at 31°C (Figure 3B). As one can see in Figure 3C, p21 expression decreased 5 fold after a 3-day culture in basal medium at 31°C and p27 expression halved. This suggests that even though a Ca^2+^/FCS switch represses the cell cycle at 37°C by inducing p21 and p27 cell cycle inhibitors, cooling at 31°C triggers the inhibition of cell cycle by a different mechanism to that observed in FCS. And at least it partially suppresses the Ca^2+^/FCS-induced p21 and p27 expression.

**Figure 4:**
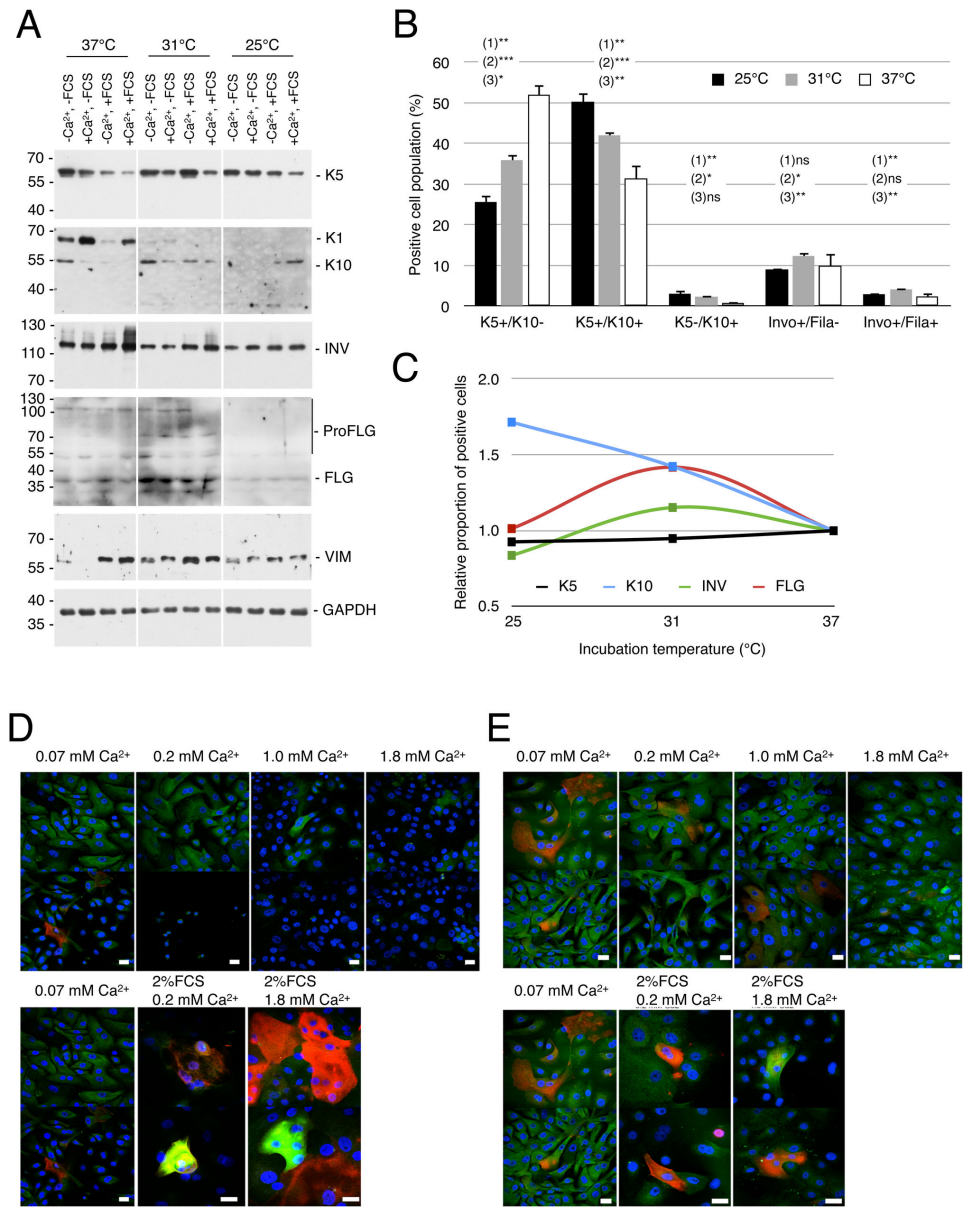
Lower incubation temperature increases keratinocyte differentiation. A. Representative immunoblotting shows detection of keratin 5 (K5), keratin 10 (K10), involucrin (INV), filaggrin (FLG), vimentin (VIM) in total protein extracts from hNEK culture with or without Ca^2+^ and FCS at 37°C, 31°C or 25°C for 4 days (n= 3). Normalization of the protein content was achieved with the GAPDH reported above. Note that monomers of FLG (36 kDa) were used as reporters of mature granular keratinocytes, though expression of bigger filaggrin precursors are shown as a control of the antibody specificity. Experiment was performed three times independently. B. Flow cytometry analysis of hNEK induced with Ca^2+^/+FCS medium at either 37°C or 31°C or 25°C for 3 days (N=3). Histogram represents the averaged percentage of cells positive for the detection of 1 or 2 differentiation markers tested by two: [K5-K10] or [INV- FLG]. Values are presented as Mean ± SD (N=3). Paired t-test were performed between (1) 37°C vs 31°C, (2) 37°C vs 25°C, (3) 31°C vs 25°C. C. Graphical representation of cold-sensitive differentiation of hNEK assessed with flow cytometry. Means (N=3) of total cells expressing K5, K10, INV or FLG at 31°C and 25°C were normalized on values at 37°C. D. Images of immunocytofluorescence show the detection of K10 (green) and INV (red) in hNEK cultured at 31°C with increasing Ca^2+^ concentrations (top panels) or with 2% FCS at different Ca^2+^ concentrations (bottom panels). Images were acquired with a confocal microscope using the setting “parameters 2” (see Material and Methods). Nuclei are counterstained with Dapi (Blue). E. same as (D) for cells grown at 25°C. Experiments presented in panels (E) and (D) were performed three times independently. Scale bars = 10 µm.

As reported in the first part of this study, a decrease of proliferation might be associated with a concomitant increase of differentiation. qPCR analysis revealed that mild cold strongly decreased *K1* expression by 3 fold, slightly reduces INV expression, did not modify *FLG* level, and triggers a 3-fold TGM1 gene upregulation. Finally, we detected that CHOP (DDIT3 gene) induction was transient at 37°C, although it was sustained at 31°C, which would suggest a more pronounced ER stress in mild cold condition than at 37°C. At the protein level, a 3-day Ca^2+^ switch was found to repress K10 expression at 37°C and 31°C (detected at 54-57 kDa; 65 kDa is likely K1 [35]), though it induced K1 at 37°C, and did not modify INV and FLG expression at 37°C and 31°C (Figure 4A). A combined mild cold treatment at 31°C with a concomitant 3-day Ca^2+^/FCS switch slightly induced FLG, strongly increased INV expression in a temperature-independent way (increased INV expression is also detected at 37°C), and decreased basal marker K5 expression. It may be noted that K10 expression in Ca^2+^/FCS medium increased as temperature decreased. In addition, cells grown at 31°C in a basal medium presented a stronger K10 and monomeric FLG expression, also suggesting an enhanced maturation of profilagrin. Conversely, at 25°C, cells lost K10 expression, except the ones grown in presence of Ca^2+^ and FCS, and showed a lower INV compared with cells grown at 31°C or 37°C. These data strongly suggested that mild cold (31°C) favors differentiation o keratinocytes induced in presence of Calcium and serum. We also confirmed that VIM expression was stimulated by FCS addition - confirming immunofluorescence data. Therefore, the apparent increase of expression of K10, INV and FLG in presence of serum would likely further increased whether we would have normalized on the proportion of keratinocytes with potency to differentiate.

We therefore went on – studying the expression of differentiation markers using flow cytometry (FC) analysis of immune-labeled cells (Figure 4B and Figure S4). The percentage of basal-like cells (K5 ^+^ /K10^-^) decreased with cooling, although the proportions of supra-basal-like cells (K5 ^+^ /K10^+^) and early spinal-like cells (K5^-^/K10^+^) increased. Besides, the proportion of late spinal-granular-like cells (INV ^+^ /FLG^-^) and granular-like cells (INV^+^/FLG^+^) increased in culture incubated at 31°C, but did not vary at 25°C. The cold-dependency of keratinocyte differentiation is presented in Figure 4C and in Figure S3B as interpolated curves representing the total number of positive cells for each differentiation markers at a given incubation temperature and normalized on this number of cells at 37°C for hNEK and HaCaT cell line respectively. We also confirmed flow cytometry data with immunofluorescence experiments, which showed a mild cold-mediated induction of both K10 and INV after a Ca^2+^/FCS switch (Figure 4D and 4E). However, one can note that K10 induction at 31°C was triggered by a small number of keratinocytes intensely expressing K10 and INV, although at 25°C K10 induction was mainly due to an increased number of keratinocytes expressing K10 at a moderate level. Altogether, these data demonstrate that mild cold (31°C) induces the expression of differentiation markers in cultured hNEK and a significant but moderate increase in the number of differentiating keratinocytes. We finally focused on hNEK expressing strong level of K10 and INV (detectable with parameters 2 of the confocal microscope) induced at 31°C.

### Mild cold stimulates a more complete granular-like differentiation of keratinocytes

**Figure 5:**
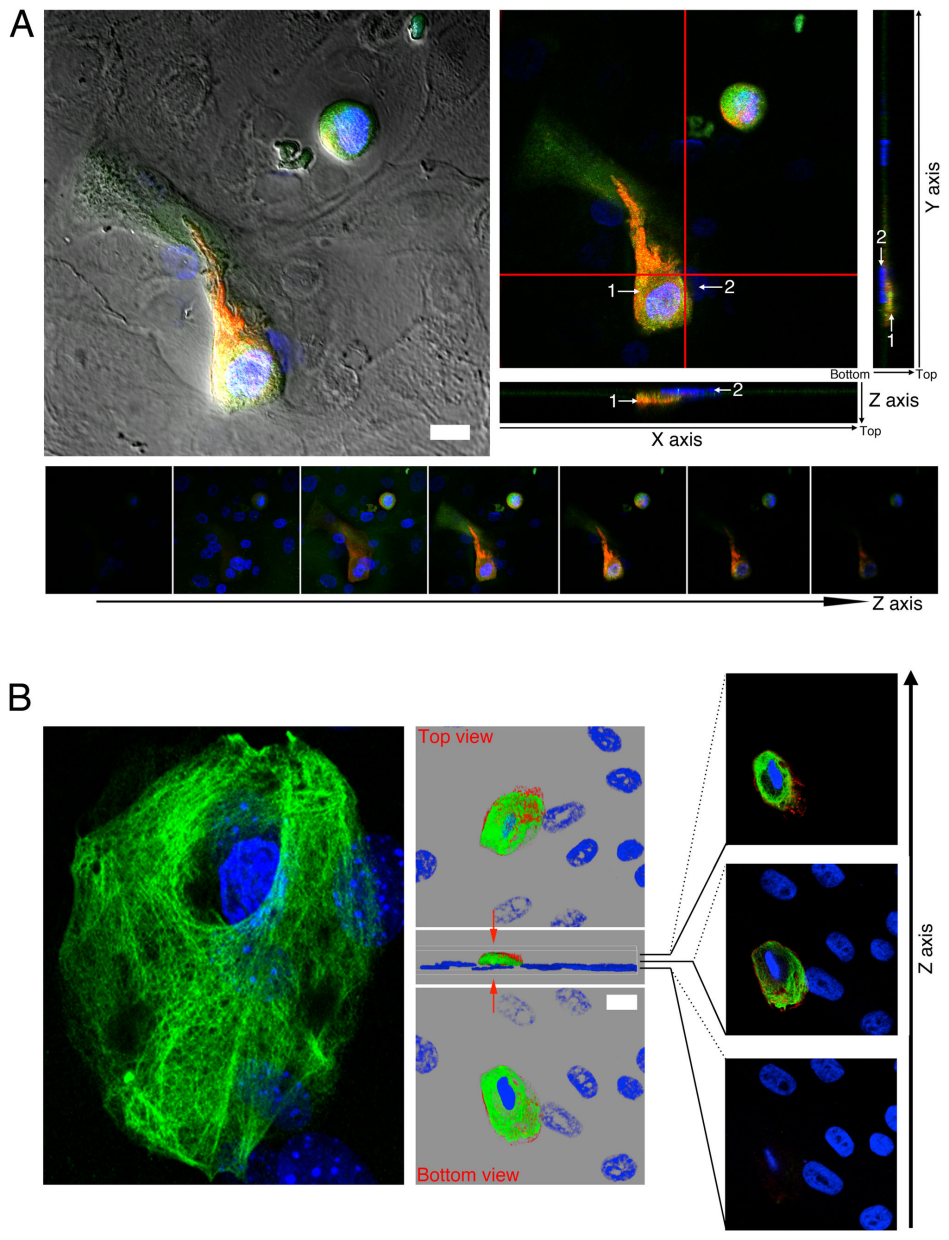
Complete Granular-like keratinocytes are rare event in *in vitro* epidermal cell culture. Granular-like keratinocytes emerged on the top of the hNEK culture and displayed expression of Involucrin and a polymerized keratin 10 network when cultured at 31°C. A. Image acquired with a LSM780 confocal microscope (parameters 2) shows detection of strong levels of K10 (green) and INV (red) in two cells emerging from a confluent culture of hNEK (top left panel). Bright field image was merged to demonstrate the confluence of the hNEK culture. Orthogonal [X-Z] and [Y-Z] views (top right panels) were performed as indicated by the red cross lines on the main picture and show that the K10 ^+^ /INV^+^ cell (cell 1) is set above a K10^-^/INV^-^ cell (cell 2) detected with its Dapi-stained nucleus. Gallery of Z-sectioning images from the bottom of the dish to the top of cell culture also shows the position of the K10 ^+^ /INV^+^ cell on the top of the cell culture. B. Confocal slide showing a polymerized network of Keratin 10 in a single keratinocyte cultured at 31°C for 5 days (left panel). 3D deconvolution (center image of middle panel) after Z-sectioning (right panel) of the same field shows the concomitant detection of K10 (green) and INV (red). Rotation of the 3D image by 90° from they axis to the Z axis shows the keratinocyte from below (bottom image of middle panel) or from above (top image of middle panel) and demonstrates a shell-like architecture of the polymerized network of K10 and INV.

We carried out immunofluorescences and performed 3D deconvolution with a confocal microscope, in order to study both the structure of the cytoskeleton in granular-like cells and the localization of these granular-like cells within the keratinocyte population. K10 ^+^ /INV^+^ keratinocytes grown at 37°C and 31°C in a Ca^2+^/FCS medium for 5 days looked flat and sat predominantly at the top of the multilayer culture (Figure 5A), whereas K10 ^+^ /INV^-^ cells were mainly located at the bottom of the dish. We reported in this study that K5 and K14 observed in basal cells displayed a polymerized network, but we never detected it for K10 in hNEK cultured at 37°C, and to our knowledge it has never been demonstrated. This is quite surprising since keratins are thought to be polymerized filaments and because Transglutaminase 1 is expected to bridge filaments between them. Strikingly, at 31°C, K10 appeared as a polymerized network forming a shell on the top and around the nucleus (Figure 5B), as expected for granular-like keratinocytes [3], and INV was associated with this K10 polymerized network. These cells were rare in our culture and represented less than 0.5% of the population. mPK grown at 31°C in a Ca^2+^/FCS medium for 5 days showed a similar pattern (Figure S5). These data suggest that the modifications of the network of secondary filaments are more efficiently achieved in keratinocytes grown at 31°C in medium complete with FCS and Ca^2+^.

## Discussion

In this study, we confirm that serum addition is required to stimulate keratinocyte differentiation at its optimal rate. We also consider that the stimulation of keratinocyte differentiation by single high Ca^2+^ switch is usually overestimated. We suggest that it is probably due to the limited number of differentiation markers and to the absence of comparison between single cell analyses and cell population analyses. Finally, we demonstrate that transient mild cold exposure optimizes the differentiation of cultured keratinocytes.

Firstly we report, at mRNA level, that the addition of 2% FCS, (including about 100 µM Ca^2+^ [36]), induces the expression of late differentiation markers more efficiently than a single high Ca^2+^ switch would. This conclusion is different from a previous study reporting a less complete differentiation with FCS than under serum-free conditions [37]. Nevertheless, our experimental conditions were stricter when measuring the serum effect, as we used the same basal medium and completed it with 2% FCS only, whereas Pillai et al compared KGM serum-free medium with DMEM supplemented with 5% FCS. Moreover, we reconciled the paradoxical effects of Ca^2+^ on differentiation marker expression by demonstrating that high Ca^2+^ switch had a permissive effect on FCS-induced late differentiation markers (INV, FLG, TGM1). We also demonstrated that an FCS switch, and to a further extent, a Ca^2+^/FCS switch, stimulates ER stress responses and up-regulates both p21 and p27 cell cycle inhibitors, thereby correlating with a previous study [29]. At the protein level, serum exerts a moderate induction of differentiation by increasing the expression of late differentiation markers.

Secondly, in agreement with previous articles [9,15,38], we report here that high Ca^2+^ switch induces a moderate up-regulation of early K1 and K10, intermediate INV and late differentiation marker TGM1 at the transcriptional level. However, we did not report a single change in the expression of other differentiation markers such as genes involved in ER stress and cell cycle. This stability of expression of cell cycle genes suggests that the lower rate of proliferation after a Ca^2+^ switch is unlikely to be correlated to a direct inhibition of the cell cycle. High Ca^2+^ level is known to trigger the formation of tight cell-to-cell interactions through desmosome completion within the timescale of 1 hour [39,40]. It is, thus, likely that this enhanced cell-to-cell interactions in turn triggers cell growth arrest. Some few studies reported an increased K10 protein expression by means of immunocytofluorescence, but as far as we know, none of them performed a concomitant detection of basal markers and none of them had tried and quantified the expression of K10 at the single cell level in order to make a comparison with its expression level in *in situ* keratinocytes. Indeed, one should expect that whether the proportion of differentiated cell rose after induction, consequently the proportion of basal cells should decrease. In this study, we demonstrated that almost 90% of hNEK expressed strong level of K5 and K14 while expressing in parallel a weak level of K10 at 37°C, which was dramatically lower than K10 level in *in situ* keratinocytes. The single high Ca^2+^ switch did not modify this proportion of cells. Furthermore, K10 detection with immunoblotting only rarely took into account the fact that its experimental size had been shown to be smaller (54-57 kDa) than the predicted one (65 kDa), thereby clearly requiring a modification in our interpretation of blots [35]. Our data demonstrates that Ca^2+^ has a permissive effect on K10 protein expression detected by immunoblotting, although it paradoxically induces K10 gene transcription. To reconcile these data, we suggest that cell population analysis is highly dependent on the background expression of proteins, which concerns the main part of cells in the case of K10 as reported with our immunocytofluorescence experiments. K10 level thus decreases in IB because of a reduction of the proportion of basal cells expressing low amount of K10 after single Ca^2+^ switch, rather than a decrease of K10 expression.

Most of the studies reporting variations in keratinocyte differentiation have used INV expression as a main differentiation marker. Accordingly to previous studies, we have found that the *INV* gene is induced quickly after Ca^2+^ switch. The associated overexpression of the INV protein has been shown to be deferred for several days after the Ca^2+^ switch [14], but this induction could also be explained by the modification of cell density with time. Indeed, INV expression is expressed in confluent keratinocytes cultures grown in basal medium. To bypass this issue, we performed experiments on keratinocyte cultures at confluence. And no significant increase of K10, INV, FLG expressions was detected at 37°C following the Ca^2+^ switch. This suggests that, though Ca^2+^ is involved at the transcription level, other mechanisms rule out the translation level of INV and K10 as well. In summary, we do not agree with the idea that single high Ca^2+^ switch is a good model for studying differentiation of *in vitro* keratinocytes. We claim here that this single high Ca^2+^ switch model is rather suitable to determine and study the Ca^2+^-dependent genes. Nevertheless, we agree that external high Ca^2+^ is an essential component of the late processes of epidermal differentiation and that it improves serum-mediated differentiation.

Thirdly, we demonstrate that keratinocyte differentiation is a cold-modulated process. As expected by the temperature-dependence of enzyme activity, we confirmed that mild cold (31°C and 25°C) induce respectively a decrease and an arrest of NEK cell growth. This decrease was not additive to the FCS-induced growth arrest, though both experimental conditions did not trigger identical molecular mechanisms. This suggests that the cell population sensitive to FCS-mediated growth arrest is the same as that one sensitive to cold-mediated growth arrest and that both stimuli trigger the maximal inhibition of cell growth. Studying the expression of genes involved in ER stress response, we detected a sustained up-regulation of CHOP after a Ca^2+^/FCS switch. This could be interpreted either as 1) as ER stress response of NEK to mild cold or 2) as a stronger induction of differentiation.

At 25°C, differentiation of keratinocytes is aborted as suggested by the cell accumulation in the supra-basal/spinal phenotype, thus demonstrating a permissive impact of this temperature on differentiation and therefore supporting the first hypothesis. However, at 31°C, the detection of a stronger K10 intensity and abnormal INV expression in cells at the base of cell culture suggests that the second hypothesis could be correct. This abnormal INV expression has been reported for reconstituted epidermis incubated at 33°C [22]. The authors concluded that supra-basal cells underwent a premature differentiation process. Moreover, we detected an increased number of keratinocytes on the top of multilayer cultures incubated at 31°C for 5 days. They expressed intense K10/INV signals, related to those observed in native human skin and native mouse skin. In these cells, K10 proteins were polymerized and organized with INV to constitute a shell-like network surrounding the nucleus. This polymerized K10 network probably reveals transglutaminase activity as expected in late spinal-like or granular-like keratinocytes. Finally, we demonstrated that monomeric FLG expression was increased in hNEK induced at 31°C, suggesting an increased maturation of the profilaggrin precursor. Altogether, these results strongly suggest that a transient exposure to mild cold enhances differentiation. However, a sustained exposure to mild cold would finally decrease the number of differentiating cells because it also inhibits the proliferation. We therefore propose that variations of temperature from 37°C to mild cold is a fine-tuning mechanism controlling the balance between proliferation and differentiation of cultured keratinocytes.

Finally, this study makes a comparison between several techniques to quantify the differentiation process. PCR and IB only show a photograph of the average gene/protein expression in a keratinocyte population. Like in every cell population analysis, the total mRNA/protein amount is a function of i) the proportion of each cell phenotypes in the cell population (keratinocytes and EMT cells), ii) the proportion of sub-phenotypes of differentiating keratinocytes (by simplification, we consider 4 major sub-phenotypes: basal, spinous, granular and corneous), and iii) the average number of differentiation markers in each phenotype. In this respect, it is uncertain whether a gene induction detected by PCR or a protein increase detected by IB is related to: i) an increase in the proportion of a marker-expressing phenotype in the population, ii) a real gene induction in a particular phenotype or iii) both previous points. It has already been reported that the percentage of differentiated keratinocytes was low and variable: less than 15% of cells were K1 and K10 positive [13], and less than 4% of cells grown in serum-free high Ca^2+^ medium supplemented with BPE were assumed to be corneocytes. One should also remember that as final transformation of keratinocyte differentiation, corneocytes are accumulating with time and their number reflects both the differentiation rate and the proliferation rate. We could therefore expect them to be less numerous at 31°C than at 37°C because of the drop in proliferation regardless of the differentiation rate. Thus, quantifying corneocytes is not adequate when we accurately measure the differentiation rate of *in vitro* keratinocytes. Moreover, according to our flow cytometry and immunocytofluorescence experiments, less than 1% of cells co-expressed K10 and INV at expression levels comparable to those in native human skin. If one assumes that culturing keratinocytes *in vitro* without feeder layer represents a good model for studying epidermal homeostasis, then the estimation of the differentiation rate should be figured out very carefully.

## Supporting Information

Figure S1 **Analysis of gene expression reveals large rearrangement after Ca^2+^/FCS induction.** Kinetics of keratinocyte differentiation reveals different serum and calcium sensitivities of gene expression. Values represent mRNA levels of the genes of interest normalized with GAPDH mRNA levels. Confluent hNEK were grown in 2% FCS + 0.1 mM Ca^2+^ for 1 day prior to the addition of 1.8 mM Ca^2+^ for 5 days (N=4). All points were significantly different from Day 0 with p<0.001, except value of P2RX7 mRNA at Day 1.
(TIF)

Figure S2 **Expression of vimentin protein in human epidermal keratinocytes.** **A**. Images were acquired with a LSM780 confocal microscope and represent the co-detection of K5(green), and VIM (red). Note that some rare cells are stained with vimentin but not with keratin 5, suggesting the presence of a weak number of fibroblast in the hNEK cultures (Scale bars = 5 µm). **B**. Immunocytofluorescence experiments show the detection of vimentin (red), in hNEK cultured (+FCS) or without (-FCS) 2% FCS and with 0.1 mM Ca^2+^ (-Ca^2+^) or 1.8 mM Ca^2+^ (+ Ca^2+^) at 37°C for 3 days. Scale bars = 10 µm. Experiments were reproduced three times independently.
(TIF)

Figure S3 **Cold-sensitivity growth and differentiation of HaCaT cell line.** A. Mild cold causes a gradual growth inhibition of the HaCaT cell line cultured in DMEM medium supplemented with 2% FCS and 0.2 mM Ca^2+^. B. Graphic representation of the cold-sensitivity of keratinocyte differentiation assessed with flow cytometry. Total cells expressing either K5 or K10 or INV or FLG at 31°C and 25°C were normalized on values at 37°C. Data are presented as mean values of three independent experiments. Smooth curves are the result of parabolic interpolation of the mean values. Although a weak tendency suggests a cold-dependent expression of differentiation markers, no statistical variations were calculated between the three temperatures of cell culturing.
(TIF)

Figure S4 **Flow cytometer data analysis of the differentiation status of hNEK.** A representative experiment shows the distribution of keratinocytes regarding on expression of either K5 and K10 or INV and FLG as reported by flow cytometry. hNEK were induced with basal KSF-SFM medium supplemented with 2% FCS, 1.8 mM Ca^2+^ at different temperatures for 3 days. Regions of interest represent: non-specific staining (black area), cell population stained with the X-axe marker and DyeLight-488 (green area), cell population stained with the Y-axe marker and AlexaFluor-647 (magenta area) and cell population stained with the two markers (cyan area). Experiment was performed three times independently.
(TIF)

Figure S5 **Cold-sensitivity of K10 expression in induced mPK.** A. K10 (green) immunocytofluorescence was performed on mouse primary keratinocytes (mPK) cultured in a basal KSF-SFM medium supplemented with 2% FCS, 1.8 mM Ca^2+^ at different temperatures (37, 31 and 25°C) for 5 days. Images of 0.9 µm thick confocal slides, acquired with Parameters 2 as described in *Materiel and methods*, shows a polymerized network of highly expressed Keratin 10 in mouse keratinocytes cultured at 31°C (middle panels). Cells expressed at 37°C or 25°C displayed a less intense and not polymerized K10 expression (up and bottom panels). Scale bars = 10 µm.
(TIF)
