# Supplementary figures and images for: Optimal Differentiation of In Vitro Keratinocytes Requires Multifactorial External Control
Source: PLoS One. 2013 Oct 7;8(10):e77507. doi: 10.1371/journal.pone.0077507 (PMC3792032; doi:10.1371/journal.pone.0077507)

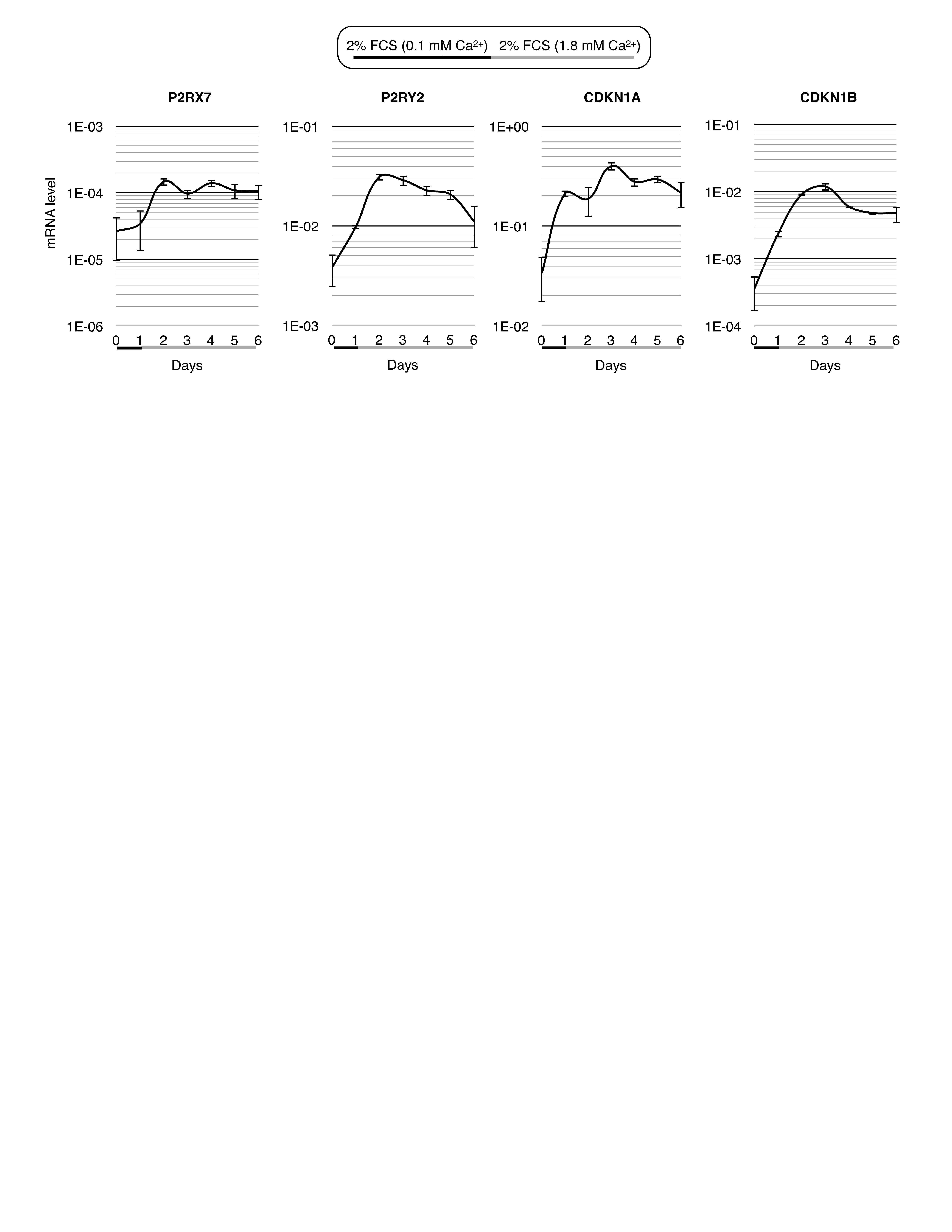

Supplement: Figure S1 — Analysis of gene expression reveals large rearrangement after Ca2+/FCS induction. Kinetics of keratinocyte differentiation reveals different serum and calcium sensitivities of gene expression. Values represent mRNA levels of the genes of interest normalized with GAPDH mRNA levels. Confluent hNEK were grown in 2% FCS + 0.1 mM Ca2+ for 1 day prior to the addition of 1.8 mM Ca2+ for 5 days (N=4). All points were significantly different from Day 0 with p<0.001, except value of P2RX7 mRNA at Day 1. (TIF) [file pone.0077507.s001.tif]

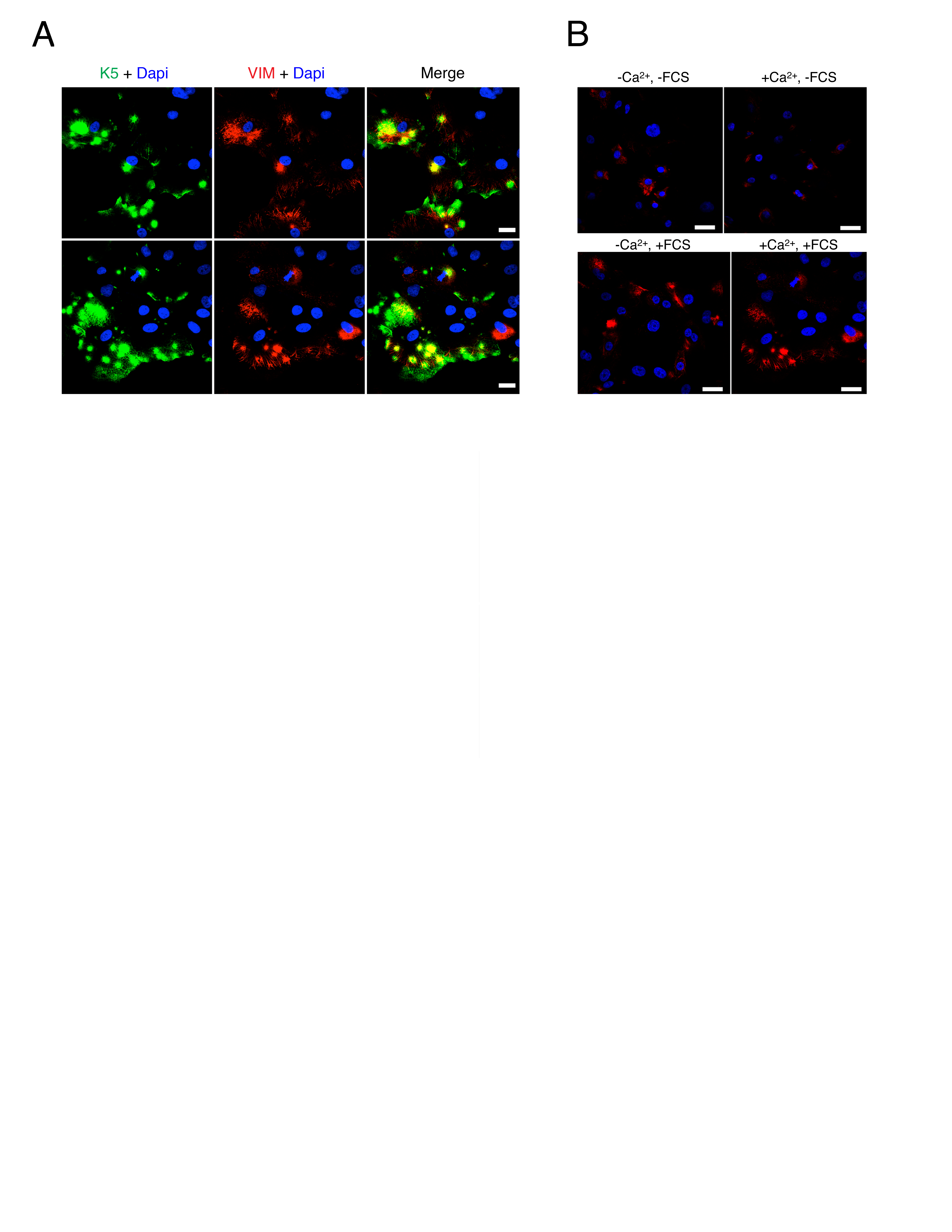

Supplement: Figure S2 — Expression of vimentin protein in human epidermal keratinocytes. A. Images were acquired with a LSM780 confocal microscope and represent the co-detection of K5(green), and VIM (red). Note that some rare cells are stained with vimentin but not with keratin 5, suggesting the presence of a weak number of fibroblast in the hNEK cultures (Scale bars = 5 µm). B. Immunocytofluorescence experiments show the detection of vimentin (red), in hNEK cultured (+FCS) or without (-FCS) 2% FCS and with 0.1 mM Ca2+ (-Ca2+) or 1.8 mM Ca2+ (+ Ca2+) at 37°C for 3 days. Scale bars = 10 µm. Experiments were reproduced three times independently. (TIF) [file pone.0077507.s002.tif]

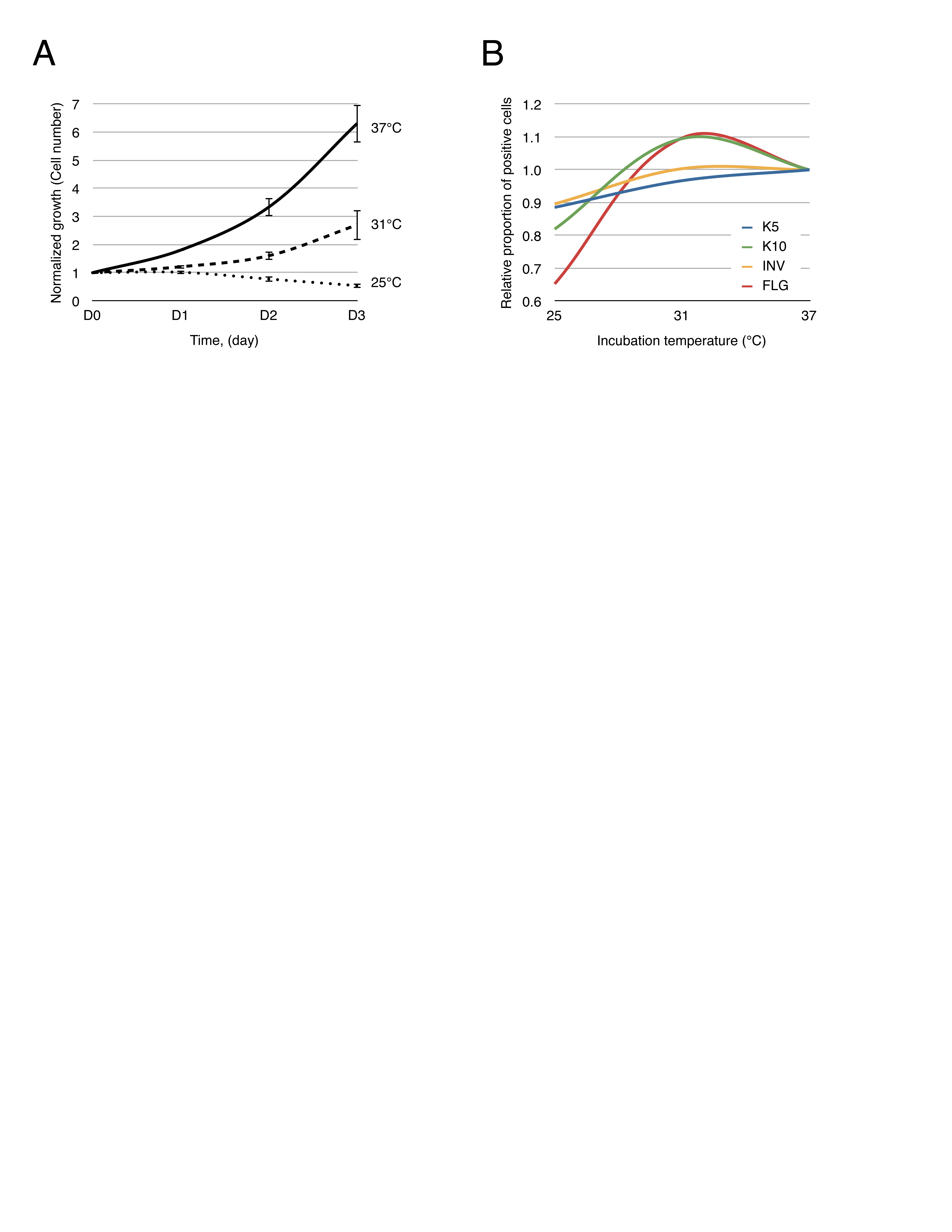

Supplement: Figure S3 — Cold-sensitivity growth and differentiation of HaCaT cell line. A. Mild cold causes a gradual growth inhibition of the HaCaT cell line cultured in DMEM medium supplemented with 2% FCS and 0.2 mM Ca2+. B. Graphic representation of the cold-sensitivity of keratinocyte differentiation assessed with flow cytometry. Total cells expressing either K5 or K10 or INV or FLG at 31°C and 25°C were normalized on values at 37°C. Data are presented as mean values of three independent experiments. Smooth curves are the result of parabolic interpolation of the mean values. Although a weak tendency suggests a cold-dependent expression of differentiation markers, no statistical variations were calculated between the three temperatures of cell culturing. (TIF) [file pone.0077507.s003.tif]

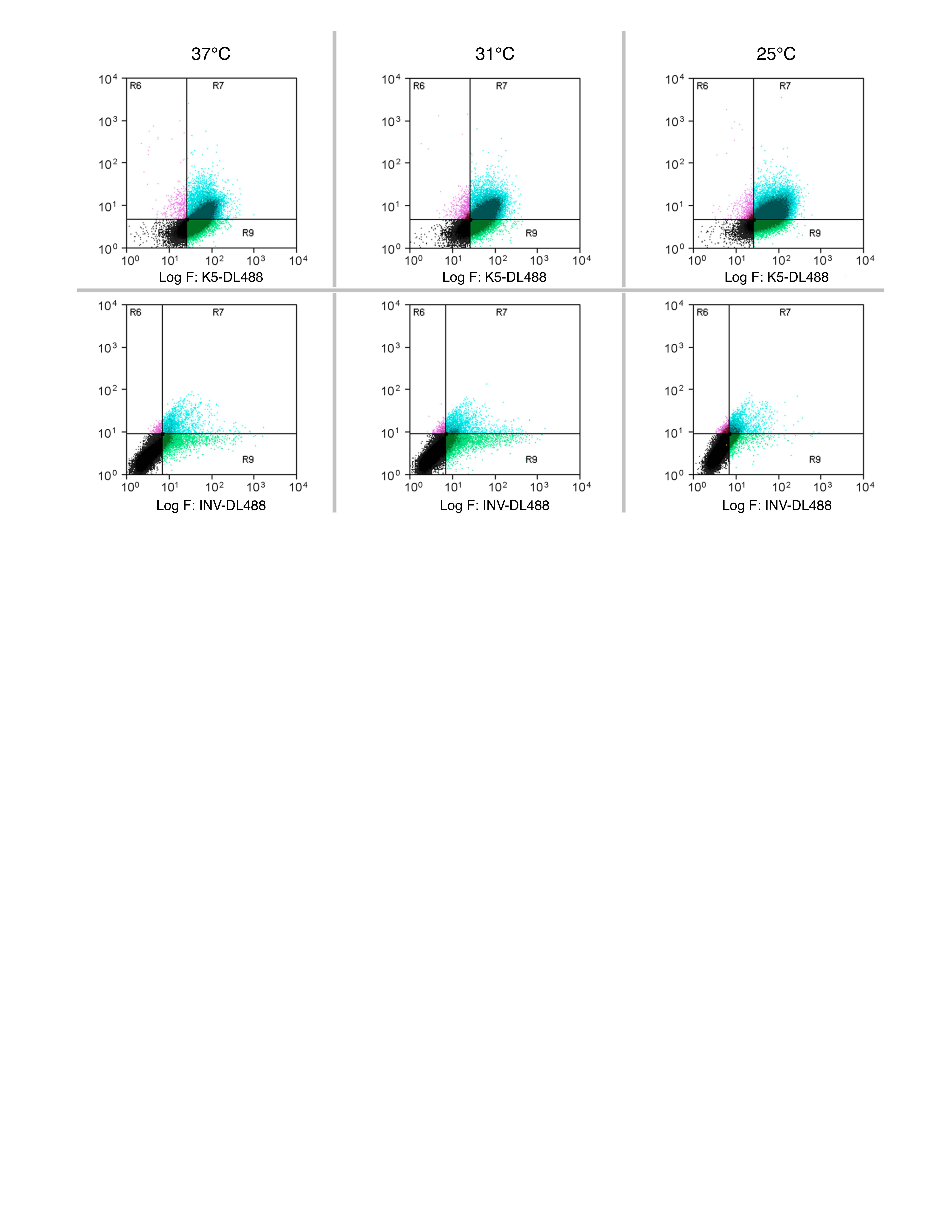

Supplement: Figure S4 — Flow cytometer data analysis of the differentiation status of hNEK. A representative experiment shows the distribution of keratinocytes regarding on expression of either K5 and K10 or INV and FLG as reported by flow cytometry. hNEK were induced with basal KSF-SFM medium supplemented with 2% FCS, 1.8 mM Ca2+ at different temperatures for 3 days. Regions of interest represent: non-specific staining (black area), cell population stained with the X-axe marker and DyeLight-488 (green area), cell population stained with the Y-axe marker and AlexaFluor-647 (magenta area) and cell population stained with the two markers (cyan area). Experiment was performed three times independently. (TIF) [file pone.0077507.s004.tif]

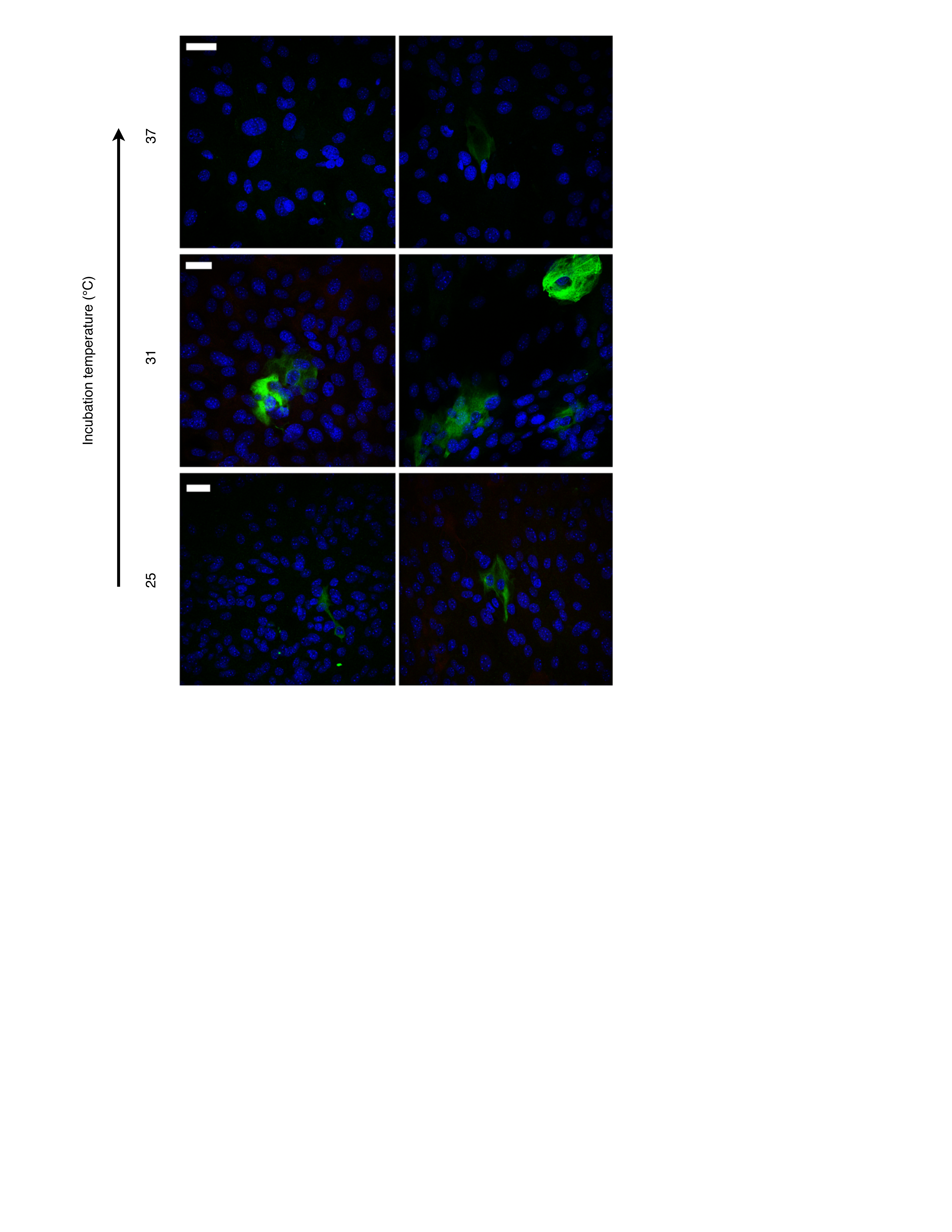

Supplement: Figure S5 — Cold-sensitivity of K10 expression in induced mPK. A. K10 (green) immunocytofluorescence was performed on mouse primary keratinocytes (mPK) cultured in a basal KSF-SFM medium supplemented with 2% FCS, 1.8 mM Ca2+ at different temperatures (37, 31 and 25°C) for 5 days. Images of 0.9 µm thick confocal slides, acquired with Parameters 2 as described in Materiel and methods, shows a polymerized network of highly expressed Keratin 10 in mouse keratinocytes cultured at 31°C (middle panels). Cells expressed at 37°C or 25°C displayed a less intense and not polymerized K10 expression (up and bottom panels). Scale bars = 10 µm. (TIF) [file pone.0077507.s005.tif]
